# Poly[(aqua­calcium)-μ_4_-pyrazine-2,3-di­carboxyl­ato]

**DOI:** 10.1107/S1600536811050276

**Published:** 2011-11-30

**Authors:** Qing-Feng Yang, Yue-Ping Zhang, Jing Lu, Ping Xue, Zheng Wang

**Affiliations:** a Key Laboratory of Energy Resources and Chemical Engineering, Ningxia University, Yinchuan 750021, Ningxia, People’s Republic of China; b School of Chemistry and Chemical Engineering, Liaocheng University, Liaocheng 252059, Shandong, People’s Republic of China

## Abstract

The polymeric title compound, [Ca(C_6_H_2_N_2_O_4_)(H_2_O)]_*n*_, was synthesized from pyrazine-2,3-dicarb­oxy­lic acid and calcium dichloride under hydro­thermal conditions. The Ca^2+^ cation is seven-coordinated by five O atoms and one N atom of four pyrazine-2,3-dicarboxyl­ate anions, and one water mol­ecule. The complete deprotonated pyrazine-2,3-dicarboxyl­ate anion adopts a μ_4_-coordination mode, resulting in the formation of a three-dimensional structure.

## Related literature

For transition and lanthanide metal complexes containing the pydc ligand (pydc = pyrazine-2,3-dicarboxyl­ate), see: Chen *et al.* (2008 ▶); Hu *et al.* (2004 ▶); Kitaura *et al.* (2002 ▶); Ma *et al.* (2006 ▶); Sakagami-Yoshida *et al.* (2000 ▶); Yin (2009 ▶); Zou *et al.* (1999 ▶).
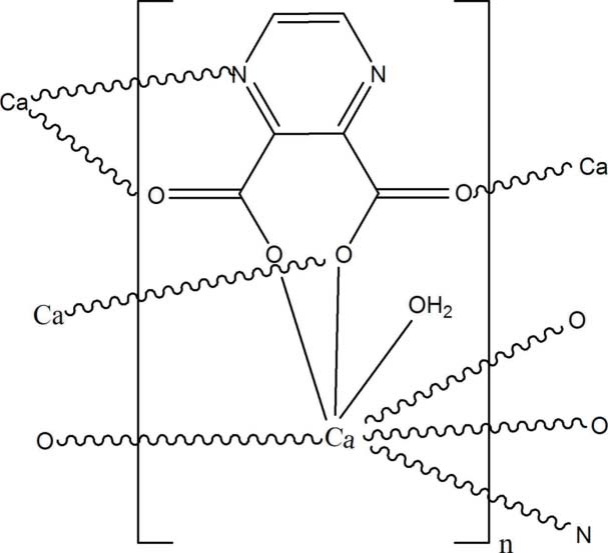


## Experimental

### 

#### Crystal data

[Ca(C_6_H_2_N_2_O_4_)(H_2_O)]
*M* *_r_* = 224.19
Monoclinic, 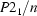

*a* = 6.8109 (7) Å
*b* = 12.0469 (13) Å
*c* = 9.9191 (11) Å
β = 102.333 (1)°
*V* = 795.08 (15) Å^3^
*Z* = 4
Mo *K*α radiation
μ = 0.79 mm^−1^
*T* = 298 K
0.35 × 0.25 × 0.10 mm

#### Data collection

Bruker SMART APEX CCD diffractometer
Absorption correction: multi-scan (*SADABS*; Bruker, 2005) ▶ *T*_min_ = 0.770, *T*_max_ = 0.926
3904 measured reflections
1405 independent reflections
1210 reflections with *I* > 2σ(*I*)
*R*_int_ = 0.026

#### Refinement

*R*[*F* ^2^ > 2σ(*F* ^2^)] = 0.028
*wR*(*F* ^2^) = 0.075
*S* = 1.06
1405 reflections
143 parameters
All H-atom parameters refined
Δρ_max_ = 0.35 e Å^−3^
Δρ_min_ = −0.34 e Å^−3^

### 

Data collection: *SMART* (Bruker, 2005 ▶); cell refinement: *SAINT* (Bruker, 2005 ▶); data reduction: *SAINT*; program(s) used to solve structure: *SHELXS97* (Sheldrick, 2008) ▶; program(s) used to refine structure: *SHELXL97* (Sheldrick, 2008) ▶; molecular graphics: *SHELXTL* (Sheldrick, 2008) ▶; software used to prepare material for publication: *SHELXL97* ▶.

## Supplementary Material

Crystal structure: contains datablock(s) I, global. DOI: 10.1107/S1600536811050276/ds2152sup1.cif

Structure factors: contains datablock(s) I. DOI: 10.1107/S1600536811050276/ds2152Isup2.hkl

Additional supplementary materials:  crystallographic information; 3D view; checkCIF report

## Figures and Tables

**Fig. 1.**
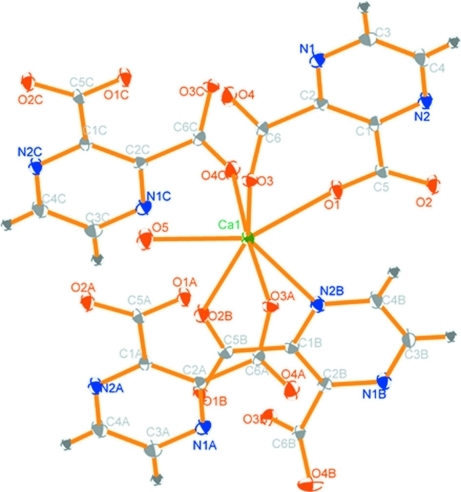
Crystal structure of compound I with labelling and displacement ellipsoids drawn at the 30% probability level. Symmetry cods: (A) -x, -y, 1 - z; (B) 1/2 - x, -1/2 + y, 2/3 - z; (C) -x, -y, 1 - z.

**Fig. 2.**
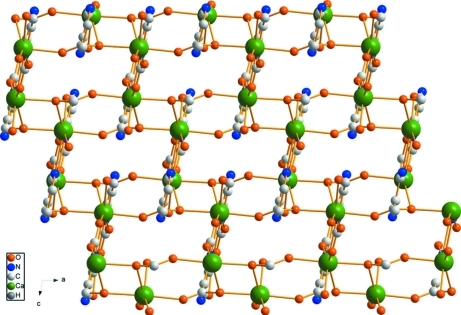
A view of the packing of compound I along b axis.

## supplementary crystallographic information

### Comment

Design and synthesis of metal-organic complexes have attracted much attention due to their intriguing molecular topologies and potentially useful properties, such as adsorption, catalytic, fluorescence, magnetic, and so on. Recently, we have learnt that pyrazine-2,3-dicarboxylic acid is an effective multifunctional bridging ligand to link the *M* ions with both N and O donor. A large number of transition and lanthanide metal complexes containing the pzdc ligand (pydc = pyrazine-2,3-dicarboxylate) have been reported, see: Zou *et al.*(1999); Sakagami-Yoshida *et al.*(2000); Kitaura *et al.*(2002); Hu *et al.*(2004); Ma *et al.*(2006); Chen *et al.*(2008); Yin *et al.*(2009). However, alkaline earth metal-containing metal-organic complexes with pydc ligand are less developed. In this paper, we report the synthesis and structure of a new Ca complex with the pzdc ligand.

The aim of the present study was to elucidate the crystal structure of the title compound, I. In I, The Ca center is seven-coordinated by five O atoms and one N atom of four deprotonated pyrazine-2,3-dicarboxylato ligands, and one water molecule (Fig. 1). The Ca—O bond lengths are between 2.3111 (14) and 2.5396 (14) Å, the Ca—N bond distance amount to 2.6159 (17) Å, (Table 1). The pyrazine-2,3-dicarboxylic acid is deprotonated completely and acted as µ_4_– ligand linking four Ca^2+^ cations. These CaO6N asymmetric units are connected *via* the anions into a three-dimensional network (Fig. 2).

### Experimental

A mixture of pyrazine-2,3-dicarboxylic acid (0.17 g, 1.00 mmol) and Calcium dichloride (0.11 g, 1.00 mmol) in distilled water (15 ml) was stirred fully in air, and then sealed in 25 ml Teflon-lined stainless steel container, which was heated at 413 K for 3 days. The Colorless block-shaped product, I, was crystallized upon cooling to 243 K.

### Refinement

All H atoms were positioned geometrically and refined using the riding-model approximation with *U*^iso^(H) = 1.5*U*^eq^(O).

### Crystal data

**Table:**

[Ca(C_6_H_2_N_2_O_4_)(H_2_O)]	*F*(000) = 456
*M**_r_* = 224.19	*D*_x_ = 1.873 Mg m^−^^3^
Monoclinic, *P*2_1_/*n*	Mo *K*α radiation, λ = 0.71073 Å
Hall symbol: -P 2yn	Cell parameters from 2583 reflections
*a* = 6.8109 (7) Å	θ = 2.7–28.2°
*b* = 12.0469 (13) Å	µ = 0.79 mm^−^^1^
*c* = 9.9191 (11) Å	*T* = 298 K
β = 102.333 (1)°	Block, colorless
*V* = 795.08 (15) Å^3^	0.35 × 0.25 × 0.10 mm
*Z* = 4	

### Data collection

**Table:**

Bruker SMART APEX CCD diffractometer	1405 independent reflections
Radiation source: fine-focus sealed tube	1210 reflections with *I* > 2σ(*I*)
graphite	*R*_int_ = 0.026
ω scans	θ_max_ = 25.0°, θ_min_ = 2.7°
Absorption correction: multi-scan (*SADABS*; Bruker, 2005)	*h* = −8→8
*T*_min_ = 0.770, *T*_max_ = 0.926	*k* = −12→14
3904 measured reflections	*l* = −10→11

### Refinement

**Table:**

Refinement on *F*^2^	Primary atom site location: structure-invariant direct methods
Least-squares matrix: full	Secondary atom site location: difference Fourier map
*R*[*F*^2^ > 2σ(*F*^2^)] = 0.028	Hydrogen site location: inferred from neighbouring sites
*wR*(*F*^2^) = 0.075	All H-atom parameters refined
*S* = 1.06	*w* = 1/[σ^2^(*F*_o_^2^) + (0.0386*P*)^2^ + 0.4192*P*] where *P* = (*F*_o_^2^ + 2*F*_c_^2^)/3
1405 reflections	(Δ/σ)_max_ < 0.001
143 parameters	Δρ_max_ = 0.35 e Å^−^^3^
0 restraints	Δρ_min_ = −0.34 e Å^−^^3^

### Special details

**Table:**

Geometry. All e.s.d.'s (except the e.s.d. in the dihedral angle between two l.s. planes) are estimated using the full covariance matrix. The cell e.s.d.'s are taken into account individually in the estimation of e.s.d.'s in distances, angles and torsion angles; correlations between e.s.d.'s in cell parameters are only used when they are defined by crystal symmetry. An approximate (isotropic) treatment of cell e.s.d.'s is used for estimating e.s.d.'s involving l.s. planes.
Refinement. Refinement of *F*^2^ against ALL reflections. The weighted *R*-factor *wR* and goodness of fit *S* are based on *F*^2^, conventional *R*-factors *R* are based on *F*, with *F* set to zero for negative *F*^2^. The threshold expression of *F*^2^ > σ(*F*^2^) is used only for calculating *R*-factors(gt) *etc*. and is not relevant to the choice of reflections for refinement. *R*-factors based on *F*^2^ are statistically about twice as large as those based on *F*, and *R*- factors based on ALL data will be even larger.

### Fractional atomic coordinates and isotropic or equivalent isotropic displacement parameters (Å^2^)

**Table:**

	*x*	*y*	*z*	*U*_iso_*/*U*_eq_	
O5	0.2040 (3)	−0.20134 (16)	0.40189 (19)	0.0390 (5)	
O2	0.2816 (2)	0.22697 (11)	0.83932 (14)	0.0279 (4)	
O4	0.4176 (2)	0.09317 (12)	0.37610 (18)	0.0343 (4)	
N2	0.2587 (3)	0.37894 (14)	0.64192 (17)	0.0218 (4)	
C4	0.2491 (4)	0.45523 (18)	0.5424 (2)	0.0268 (5)	
Ca1	0.23561 (6)	−0.08343 (3)	0.59754 (4)	0.01641 (15)	
O3	0.1145 (2)	0.06496 (11)	0.41707 (14)	0.0207 (3)	
O1	0.3116 (2)	0.08934 (10)	0.69619 (14)	0.0256 (4)	
C6	0.2677 (3)	0.12378 (16)	0.41813 (19)	0.0170 (4)	
C1	0.2679 (3)	0.27200 (15)	0.60607 (19)	0.0173 (4)	
C5	0.2868 (3)	0.18869 (15)	0.72322 (19)	0.0181 (4)	
C2	0.2662 (3)	0.24255 (16)	0.46930 (19)	0.0171 (4)	
N1	0.2632 (3)	0.31989 (14)	0.37173 (17)	0.0238 (4)	
C3	0.2526 (4)	0.42579 (17)	0.4091 (2)	0.0284 (5)	
H2	0.242 (3)	0.526 (2)	0.564 (2)	0.024 (6)*	
H1	0.245 (3)	0.479 (2)	0.336 (2)	0.029 (6)*	
H3	0.212 (5)	−0.198 (3)	0.319 (4)	0.072 (11)*	
H4	0.207 (5)	−0.260 (3)	0.426 (4)	0.073 (12)*	

### Atomic displacement parameters (Å^2^)

**Table:**

	*U*^11^	*U*^22^	*U*^33^	*U*^12^	*U*^13^	*U*^23^
O5	0.0702 (13)	0.0292 (10)	0.0187 (9)	−0.0027 (9)	0.0122 (8)	−0.0006 (7)
O2	0.0484 (10)	0.0199 (8)	0.0161 (7)	0.0005 (7)	0.0082 (7)	−0.0016 (6)
O4	0.0211 (9)	0.0356 (9)	0.0487 (10)	−0.0014 (7)	0.0130 (7)	−0.0179 (7)
N2	0.0281 (10)	0.0171 (8)	0.0193 (9)	0.0012 (7)	0.0034 (7)	−0.0005 (7)
C4	0.0397 (14)	0.0160 (10)	0.0243 (11)	0.0017 (9)	0.0062 (10)	0.0023 (9)
Ca1	0.0167 (2)	0.0162 (2)	0.0158 (2)	−0.00003 (14)	0.00219 (16)	−0.00004 (14)
O3	0.0178 (7)	0.0215 (7)	0.0225 (7)	−0.0037 (6)	0.0035 (6)	−0.0001 (6)
O1	0.0408 (9)	0.0156 (7)	0.0190 (8)	0.0019 (6)	0.0031 (7)	−0.0007 (5)
C6	0.0179 (11)	0.0208 (10)	0.0110 (9)	0.0009 (8)	0.0002 (8)	0.0008 (8)
C1	0.0176 (10)	0.0175 (10)	0.0164 (10)	0.0002 (8)	0.0028 (8)	−0.0012 (8)
C5	0.0192 (10)	0.0171 (10)	0.0170 (10)	−0.0008 (8)	0.0016 (8)	−0.0019 (8)
C2	0.0144 (10)	0.0202 (10)	0.0162 (10)	−0.0013 (8)	0.0023 (8)	0.0003 (8)
N1	0.0292 (10)	0.0233 (9)	0.0184 (8)	−0.0005 (7)	0.0043 (7)	0.0016 (7)
C3	0.0422 (14)	0.0208 (11)	0.0215 (12)	−0.0003 (9)	0.0055 (10)	0.0059 (9)

### Geometric parameters (Å, °)

**Table:**

O5—Ca1	2.3774 (17)	Ca1—O2^iv^	2.3779 (14)
O5—H3	0.83 (4)	Ca1—O3	2.5396 (14)
O5—H4	0.74 (4)	Ca1—N2^iv^	2.6159 (17)
O2—C5	1.248 (2)	Ca1—Ca1^iii^	3.9284 (8)
O2—Ca1^i^	2.3779 (14)	Ca1—H4	2.70 (4)
O4—C6	1.238 (2)	O3—C6	1.260 (2)
O4—Ca1^ii^	2.3233 (15)	O3—Ca1^iii^	2.3682 (14)
N2—C4	1.340 (3)	O1—C5	1.246 (2)
N2—C1	1.341 (3)	C6—C2	1.519 (3)
N2—Ca1^i^	2.6159 (17)	C1—C2	1.400 (3)
C4—C3	1.374 (3)	C1—C5	1.520 (3)
C4—H2	0.88 (2)	C2—N1	1.340 (2)
Ca1—O1	2.3111 (14)	N1—C3	1.335 (3)
Ca1—O4^ii^	2.3233 (15)	C3—H1	0.96 (2)
Ca1—O3^iii^	2.3682 (14)		
			
Ca1—O5—H3	139 (2)	O3^iii^—Ca1—Ca1^iii^	38.35 (3)
Ca1—O5—H4	108 (3)	O5—Ca1—Ca1^iii^	88.95 (5)
H3—O5—H4	112 (3)	O2^iv^—Ca1—Ca1^iii^	122.70 (4)
C5—O2—Ca1^i^	127.64 (12)	O3—Ca1—Ca1^iii^	35.36 (3)
C6—O4—Ca1^ii^	150.28 (14)	N2^iv^—Ca1—Ca1^iii^	114.42 (4)
C4—N2—C1	117.46 (18)	O1—Ca1—H4	162.6 (8)
C4—N2—Ca1^i^	126.65 (14)	O4^ii^—Ca1—H4	88.1 (7)
C1—N2—Ca1^i^	115.89 (12)	O3^iii^—Ca1—H4	95.7 (7)
N2—C4—C3	121.6 (2)	O5—Ca1—H4	15.2 (8)
N2—C4—H2	118.6 (14)	O2^iv^—Ca1—H4	53.8 (8)
C3—C4—H2	119.8 (14)	O3—Ca1—H4	98.1 (8)
O1—Ca1—O4^ii^	82.39 (5)	N2^iv^—Ca1—H4	117.9 (8)
O1—Ca1—O3^iii^	94.17 (5)	Ca1^iii^—Ca1—H4	98.7 (8)
O4^ii^—Ca1—O3^iii^	176.20 (5)	C6—O3—Ca1^iii^	140.29 (13)
O1—Ca1—O5	148.96 (6)	C6—O3—Ca1	104.26 (11)
O4^ii^—Ca1—O5	88.53 (7)	Ca1^iii^—O3—Ca1	106.29 (5)
O3^iii^—Ca1—O5	95.26 (6)	C5—O1—Ca1	158.12 (14)
O1—Ca1—O2^iv^	140.60 (5)	O4—C6—O3	124.80 (18)
O4^ii^—Ca1—O2^iv^	91.60 (6)	O4—C6—C2	117.25 (17)
O3^iii^—Ca1—O2^iv^	90.05 (5)	O3—C6—C2	117.90 (16)
O5—Ca1—O2^iv^	68.91 (6)	N2—C1—C2	120.60 (17)
O1—Ca1—O3	70.98 (5)	N2—C1—C5	115.63 (16)
O4^ii^—Ca1—O3	106.51 (5)	C2—C1—C5	123.74 (17)
O3^iii^—Ca1—O3	73.71 (5)	O1—C5—O2	125.83 (18)
O5—Ca1—O3	83.45 (6)	O1—C5—C1	117.58 (17)
O2^iv^—Ca1—O3	146.61 (5)	O2—C5—C1	116.55 (16)
O1—Ca1—N2^iv^	77.47 (5)	N1—C2—C1	121.28 (18)
O4^ii^—Ca1—N2^iv^	94.59 (6)	N1—C2—C6	114.43 (16)
O3^iii^—Ca1—N2^iv^	83.05 (5)	C1—C2—C6	124.29 (17)
O5—Ca1—N2^iv^	133.04 (6)	C3—N1—C2	117.23 (17)
O2^iv^—Ca1—N2^iv^	64.18 (5)	N1—C3—C4	121.8 (2)
O3—Ca1—N2^iv^	138.80 (5)	N1—C3—H1	115.3 (14)
O1—Ca1—Ca1^iii^	80.40 (4)	C4—C3—H1	122.9 (14)
O4^ii^—Ca1—Ca1^iii^	141.75 (5)		

Symmetry codes: (i) −*x*+1/2, *y*+1/2, −*z*+3/2; (ii) −*x*+1, −*y*, −*z*+1; (iii) −*x*, −*y*, −*z*+1; (iv) −*x*+1/2, *y*−1/2, −*z*+3/2.
